# Bioinspired anisotropic surfaces with hybrid wettability for enhanced fog harvesting

**DOI:** 10.1039/d6ra06775a

**Published:** 2026-09-08

**Authors:** Xiangge Bai, Yishan Chen, Jiahui Chu, Lemin Zhang, Yahua Liu

**Affiliations:** a State Key Laboratory of High-performance Precision Manufacturing, Dalian University of Technology Dalian 116024 P. R. China yahualiu@dlut.edu.cn

## Abstract

Water scarcity poses a growing global challenge, and atmospheric water harvesting has emerged as a promising decentralized solution. Bioinspired surfaces with hybrid wettability have shown potential for fog collection, yet most existing designs suffer from trade-offs between droplet condensation and removal, limiting their overall collection efficiency and long-term durability under real-world conditions. Moreover, the integration of anisotropic droplet transport to accelerate surface refreshment remains underexplored in a single, scalable platform. Inspired by the dorsal bumps of desert beetles and the micro-grooved structures of rice leaves, here we fabricate a bioinspired surface featuring both hybrid wettability and structural anisotropy (HWSA) using accessible electrochemical deposition, hydrophobic modification, and spray-coating techniques. The protruding hydrophilic particles accelerate droplet nucleation and growth, while the anisotropic hydrophobic background promotes rapid, directional droplet shedding to continuously refresh the collection area. Consequently, the HWSA surface achieves a maximum fog-collection rate of 4.3 g cm^−2^ h^−1^ and retains stable performance across 20 operational cycles. Furthermore, the surface displays exceptional self-cleaning capabilities, offering a durable and scalable solution for practical atmospheric water harvesting in resource-scarce environments.

## Abstract 2

A bioinspired fog-collection surface with hybrid wettability and structural anisotropy was developed by mimicking desert beetle bumps and rice-leaf grooves, enabling rapid droplet growth and directional shedding.

## Graphical abstract

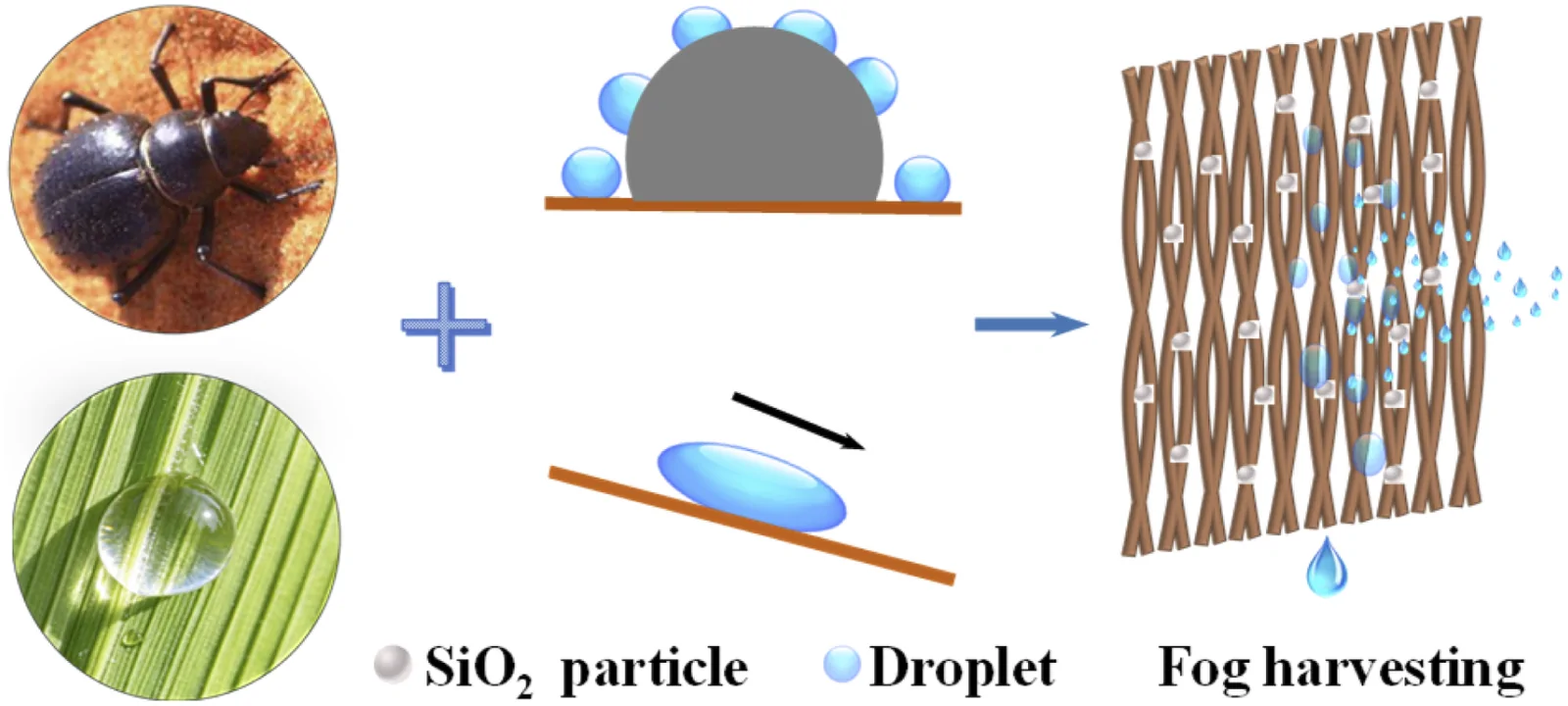

## 1 Introduction

Although Earth holds abundant water resources, the severe shortage of accessible freshwater for human consumption and industrial use poses a growing threat to global sustainable development and ecological security.^1–3^ Various approaches have been employed to obtain clean water,^4–7^ yet atmospheric fog—accounting for approximately 10% of total freshwater on Earth^8^—represents an underutilized and promising source. Harvesting fog from the atmosphere thus offers a viable path to alleviating the freshwater crisis. Numerous fog collectors have been developed to this end, including field-drive systems^9^ and harp-shaped designs,^10,11^ but these suffer from limited efficiency and high fabrication costs.

In nature, numerous organisms have evolved specialized surface topographies and wettability characteristics to adapt to harsh environments. Notable examples include the hybrid wettability patterns on desert beetle elytra,^12^ the spiky structure of cacti,^13^ the spindle-knot architecture of spider silk,^14^ hydrophilic groove channel on bat tongues^15^ and the microgrooves on rice leaves.^16^ Inspired by these natural features, researchers have developed various hybrid-wettability fog collectors.^17^ For example, hybrid cellulose membranes have been prepared,^18^ yet they suffer from uneven hydrophilic–hydrophobic distribution. Similarly, Wen *et al.* fabricated a fog collector comprising a hydrophobic substrate with hydrophilic needle arrays,^19^ which enables droplet capture but relies solely on gravity for droplet removal, thereby limiting overall efficiency. Moreover, existing fabrication techniques for super-wettability surfaces—including lithography,^20–22^ chemical vapor deposition,^23^ laser ablation,^24–26^ wire-cutting,^27^—are costly, complex, and time-consuming. An efficient fog collection process relies on three sequential stages: droplet capture/condensation, coalescence/growth, and rapid shedding.^28^ Therefore, developing a simple, cost-effective method to fabricate surfaces that facilitate both rapid droplet capture and accelerated shedding is essential.

In this work, an anisotropic, biomimetic surface with hybrid wettability was fabricated *via* a straightforward three-step process. A superhydrophobic, micro-grooved substrate was prepared on stainless steel mesh *via* electrochemical deposition and thiolation self-assembly, mimicking the hydrophobic channels of desert beetles and the anisotropic grooves of rice leaves. Subsequently, hydrophilic SiO_2_ nanoparticles were spray-coated onto the substrate to form uniformly distributed hydrophilic bumps, mimicking the beetle's water-capturing nodes. The hydrophilic SiO_2_ bumps accelerate droplet condensation and nucleation, while the underlying anisotropic hydrophobic mesh promotes rapid droplet departure, yielding a high fog collection efficiency of 4.3 g cm^−2^ h^−1^. Furthermore, the surface maintained stable efficiency over 20 collection cycles. The proposed fabrication strategy is simple, low-cost, and scalable, offering strong potential for practical atmospheric water harvesting.

## 2 Experimental

### 2.1 Materials

Stainless steel mesh (2000 mesh size, SSM) was purchased from Shanghai Yanjin wire mesh manufacturer. Copper acetate (Cu(Ac)_2_) was obtained from Tianjin Tianli Chemical Reagent Co., Ltd. Sodium acetate (NaAc) was supplied by Ron's Reagent. Dodecanethiol (CH_3_(CH)_2_SH) was purchased from Shanghai Aladdin Biochemical technology Co., Ltd, China. Ethanol was provided by Tianjin Fuyu Fine Chemical Co. Ltd. Silicon dioxide (SiO_2_) nanoparticles and acetone were purchased from Liaodong Reagent Factory. A saturated calomel electrode (SCE) was used as supplied.

### 2.2 Preparation of hybrid wettability SSM

SSM sheets were cut into 3 × 3 cm squares and ultrasonically cleaned in acetone and ethanol sequentially for 5 min to remove surface impurities. The cleaned mesh was then vertically immersed in an electrolyte solution containing 0.02 M Cu (Ac)_2_ and 0.1 M NaAc. Cu_2_O was electrodeposited onto the SSM using a three electrode electrochemical workstation, with a polished copper block as the counter electrode and a saturated calomel electrode as the reference electrode. The working electrode was positioned parallel to the counter electrode at a distance of 2 cm. Electrodeposition was performed at −0.7 V for 15 min, after which the SSM surface was rinsed thoroughly with distilled water to remove residual electrolyte. The reaction proceeds as follows

1 Cu^2+^ + 2e^−^ → Cu

2 2Cu^2+^ + 2e^−^ + H_2_O → Cu_2_O + 2H^+^

To achieve superhydrophobicity, the Cu_2_O-coated SSM was immersed in a dodecanethiol/ethanol solution (1 : 10 V/V) for 30 min

3 2CH_3_(CH_2_)_11_SH + Cu_2_O → 2CH_3_(CH_2_)_11_SCu + H_2_O

Finally, an SiO_2_ nanoparticle suspension was sprayed onto the superhydrophobic Cu_2_O-coated SSM to achieve uniform particle distribution. Among them, the spraying pressure and distance were 0.2 MPa and 15 cm, respectively. The whole process took approximately 5 min. The surface was then rinsed with ethanol and dried in an oven to obtain the final HWSA surface. For comparison, superhydrophilic and superhydrophobic Cu_2_O surfaces were also prepared *via* electrochemical deposition, with the latter receiving an additional hydrophobic treatment.

### 2.3 Characterization

Surface morphology and elemental distribution were analyzed using Scanning Electron Microscopy (SEM, HITACHI, SU5000, Japan) equipped with an energy dispersive spectrometer (EDS). Surface chemical composition was examined by X-ray photoelectron spectroscopy (XPS, Thermo Fisher, K-Alpha+, Britain). Water contact angles were measured at room temperature using an optical contact angle goniometer (Dataphysics, OCA25, German) with 3 µL water droplets. Five independent measurements were performed at different locations on each substrate. Microscopic and macroscopic fog collection behaviors were captured using an ultra-depth-of-field microscope and a digital camera, respectively. Three-dimensional surface topographies were recorded using a non-contact 3D surface profiler (ZYGO, NV5022, Middlefield, CT, USA).

### 2.4 Fog collection experiment

Fog collection tests were conducted at 25 °C. The sample was mounted vertically on a frame positioned 10 cm from the nozzle of a commercial ultrasonic humidifier (Deerma F628), which generated a fog flow velocity of approximately 50 cm s^−1^. A beaker was placed directly beneath the sample to collect runoff water. Control samples included SHLM and SHBM, and a hybrid-wettability surface fabricated on a flat stainless-steel sheet (HWS). The fog collection efficiency (*η*) was calculated using the following equation4
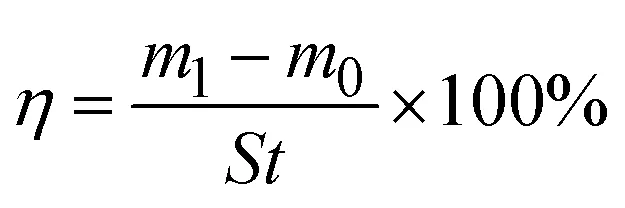
where *m*_1_ is the final mass of the beaker after collecting, *m*_0_ is the initial mass of the empty beaker, *S* is the projected surface area of the sample, and *t* is the collection duration.

## 3 Results and discussion

**Fig. 1:**
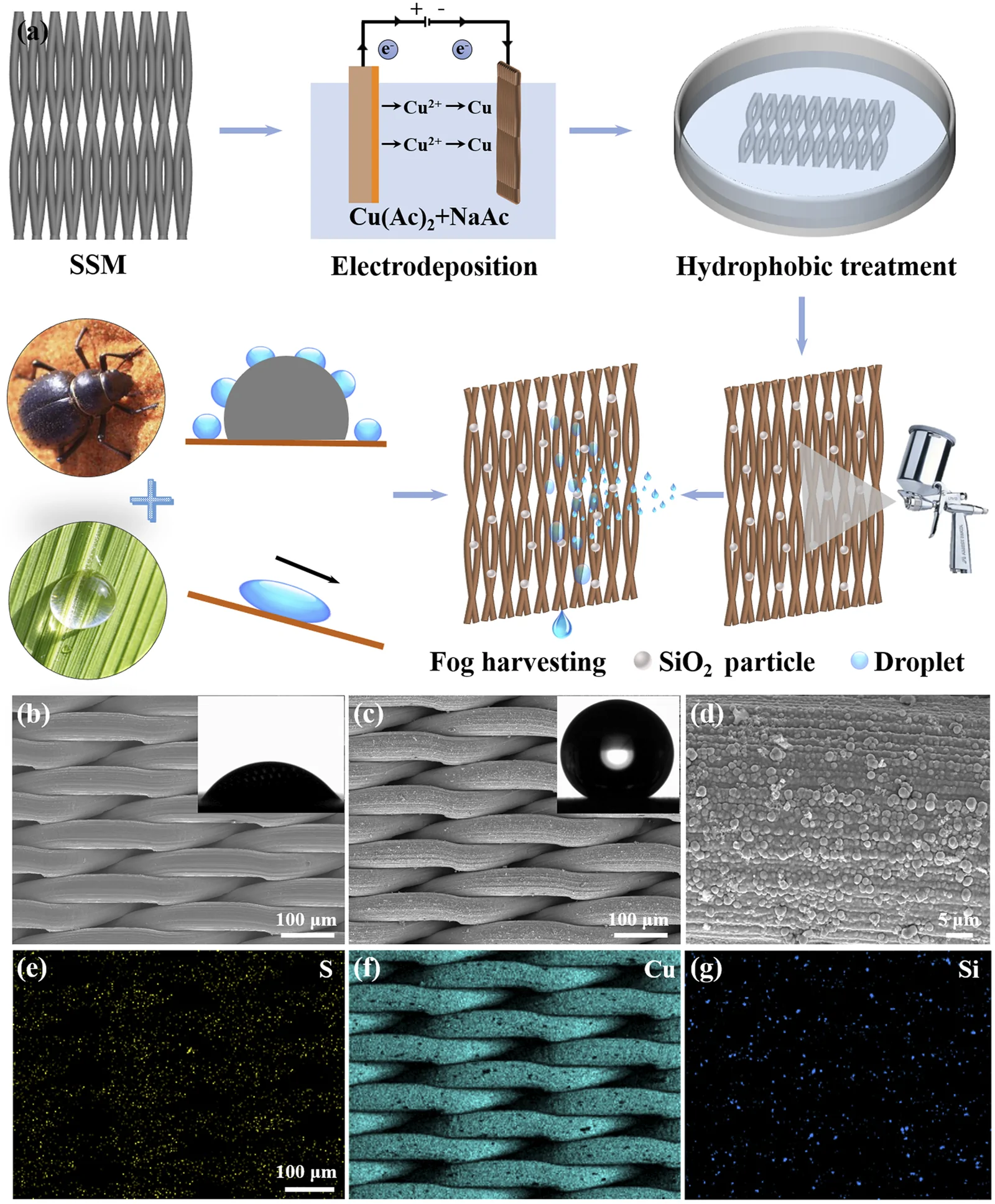
(a) Schematic diagram of the preparation process of the HWSA surface. (b) FE-SEM images of the original SSM. Images of (c) FE-SEM and (d) high-magnification FE-SEM of the HWSA surface. The insets show the contact angle. EDS analysis corresponding elemental maps for (e) S, (f) Cu and (g) Si.

**Fig. 2:**
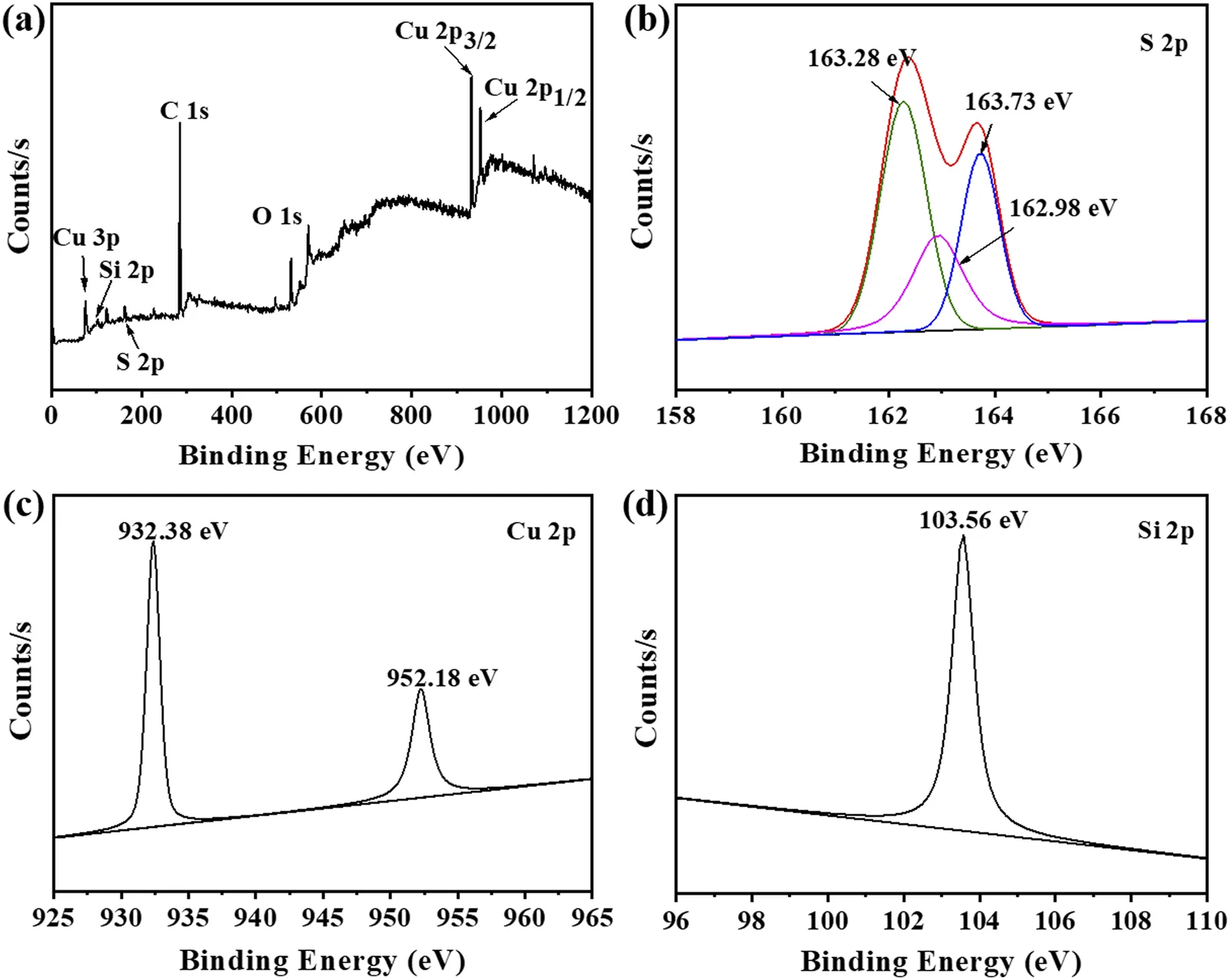
(a) XPS survey spectrum of the HWSA surface. High-resolution XPS spectra of (b) S, (c) Cu and (d) Si.

The fabrication process of the HWSA surface is schematically illustrated in Fig. 1a. The surface comprises a superhydrophobic SSM substrate decorated with uniformly distributed superhydrophilic SiO_2_ nanoparticles. This spatial arrangement mimics the dual-wettability architecture of desert beetle elytra, promoting rapid droplet nucleation. Simultaneously, the woven mesh structure imparts structural anisotropy similar to that of rice leaves, facilitating directional droplet drainage. While the original SSM was relatively smooth (Fig. 1b) and moderately hydrophobic, with a contact angle of 53° (Inset of Fig. 1b), the fabricated HWSA surface displayed a micro/nanostructured rough topography (Fig. 1c and d) and superhydrophobic properties, achieving a water contact angle of 155° (Inset of Fig. 1c). Elemental mapping *via* EDS confirmed that Cu and S signals covered the underlying mesh framework, while Si signals corresponding to SiO_2_ nanoparticles were uniformly distributed across the surface (Fig. 1e and f). Surface chemical composition were measured by XPS. As shown in Fig. 2a, multiple element of S, Cu, O, C, and Si can be observed. Further analysis was conducted on high-resolution XPS spectra of S 2p, Cu 2p, and Si 2p. The S 2p peak at 162.28 eV is related to sulfur of C_12_H_25_SCu, 163.73 eV is attributed to sulfur of the unbound thiols, and the S 2p peak at 162.93 eV is attributed to disulfide during the reaction of Cu_2_O with dodecanethiol (Fig. 2b). The XPS spectrum of Cu 2p showed that the Cu 2p_1/2_ and 2p_3/2_ peaks centered at binding energies of 932.8 and 952.8 eV can be assigned to Cu^+^ species (Fig. 2c).^29^ The high-resolution XPS spectra of Si 2p show peaks Si–O–Si (103.2 eV) due to the SiO_2_ (Fig. 2d).^30^ High-resolution XPS spectra confirmed the presence of copper dodecanethiolate Cu(S(CH_2_)_11_CH_3_)_2_ and SiO_2_, verifying successful surface functionalization.^31,32^

**Fig. 3:**
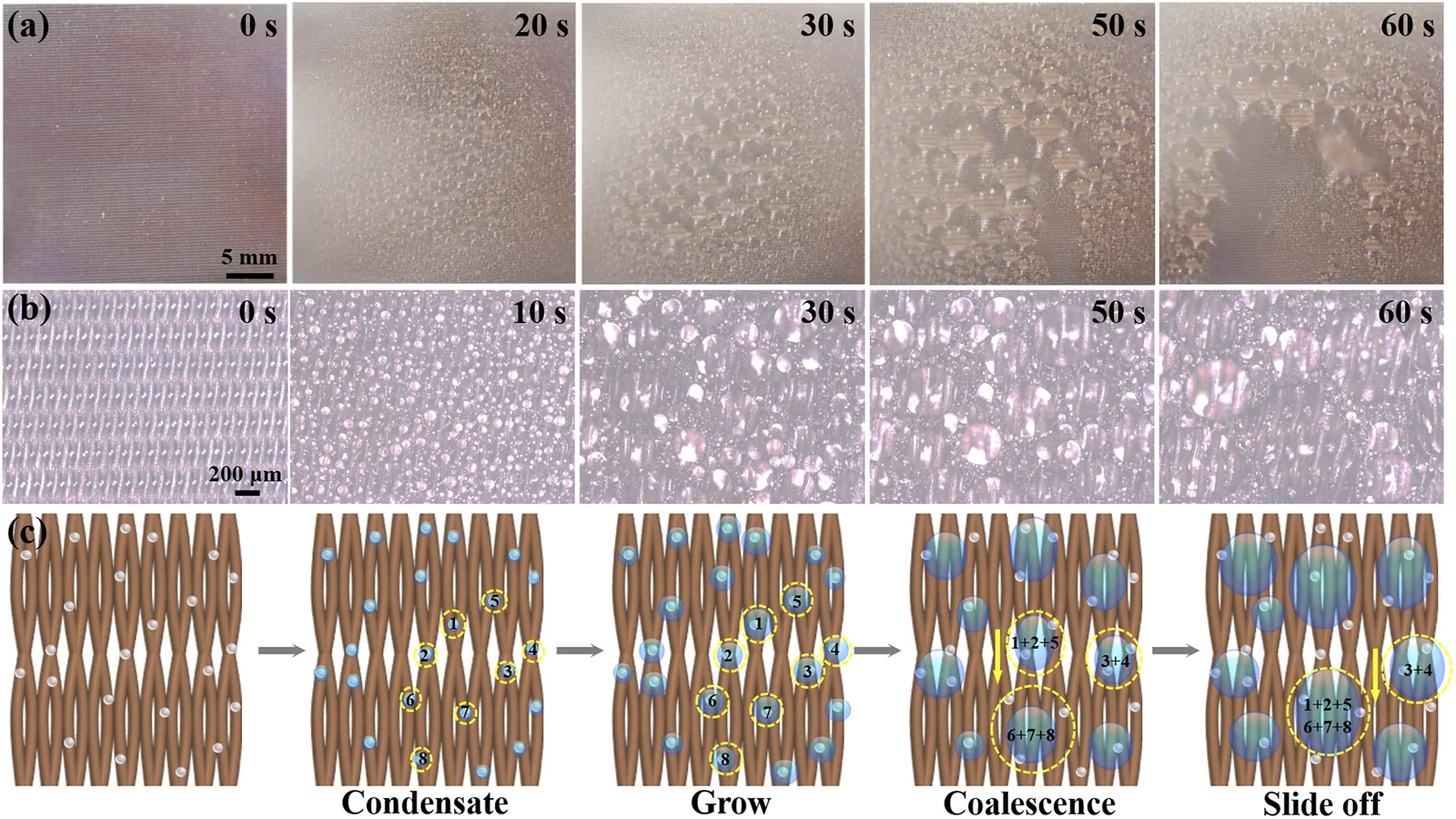
(a) Macroscopic and (b) microscopic fog collection processes on the HWSA surface. (c) Schematic illustration of the fog collection mechanism, detailing droplet condensation, growth, coalescence, and shedding.

Macroscopic and microscopic condensation dynamics were monitored in real time. Macroscopically (Fig. 3a), microdroplets continuously nucleated, coalesced into larger droplets, and rapidly shed from the surface under gravity. Microscopically (Fig. 3b), small droplets nucleated along the woven ridges within 10 s of fog exposure, grew along the directional channels of the mesh, and merged across adjacent wire grooves. Upon reaching a critical size, the droplets rapidly drained along these anisotropic channels. Fig. 3c summarizes the mechanistic pathway: hydrophilic SiO_2_ sites can effectively capture fog droplets and facilitate their subsequent growth and coalescence, while the surrounding anisotropic superhydrophobic channels promote rapid droplet growth and directional shedding.

**Fig. 4:**
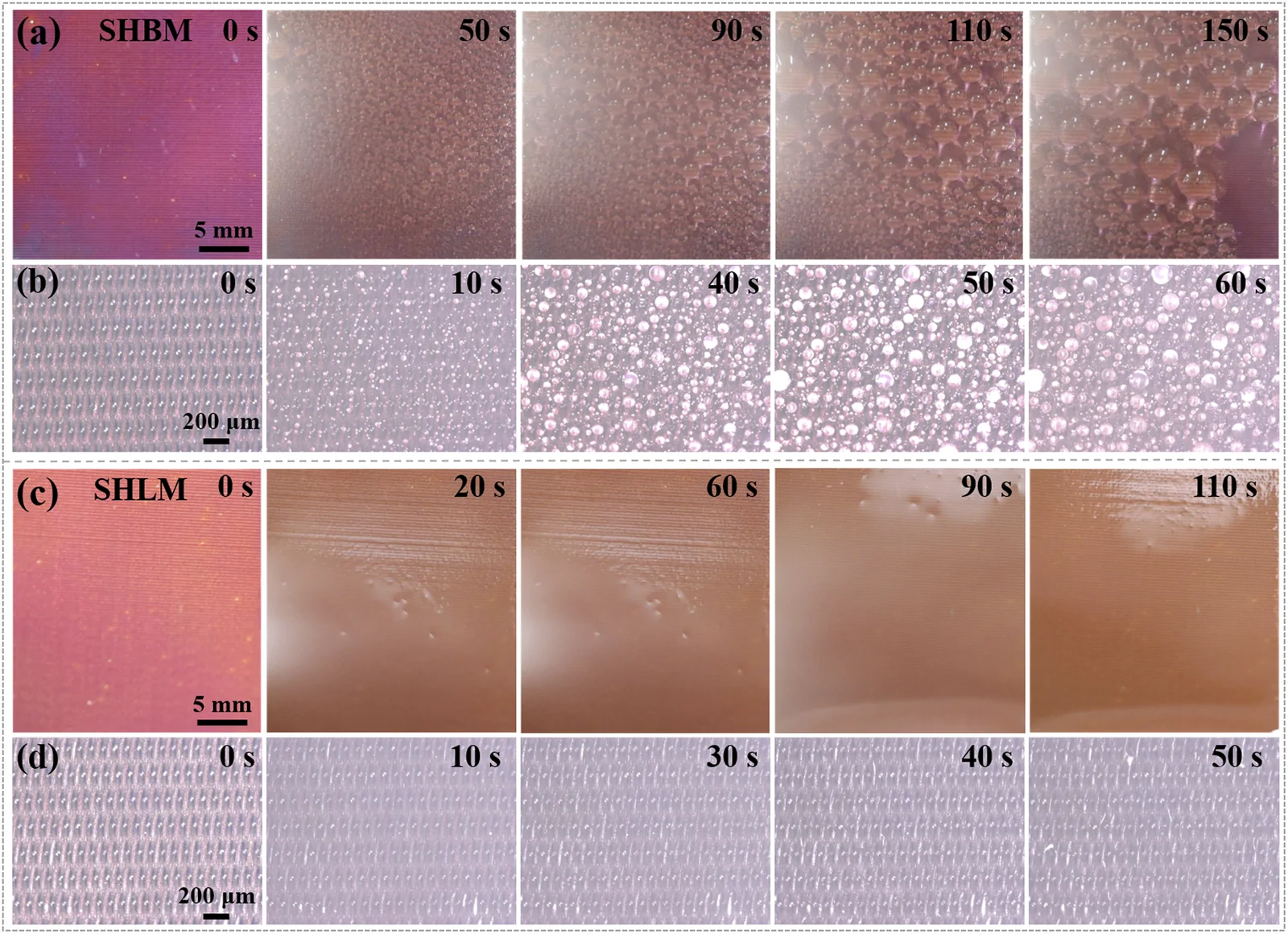
Macroscopic (a and c) and microscopic (b and d) fog collection on SHBM and SHLM surfaces.

To highlight the advantage of hybrid wettability, we compared droplet behavior on SHBM and SHLM surfaces. On the SHBM (Fig. 4a and b), numerous tiny droplets form and grow, but coalescence is limited; most remain isolated, preventing an efficient condensation–detachment cycle. Conversely, on SHLM (Fig. 4c and d), droplets immediately spread into a continuous film that thickens over time, forming a retained water layer that suppresses further nucleation and growth; only the woven substrate structure is visible, with no directional droplet sliding, markedly reducing collection efficiency.

**Fig. 5:**
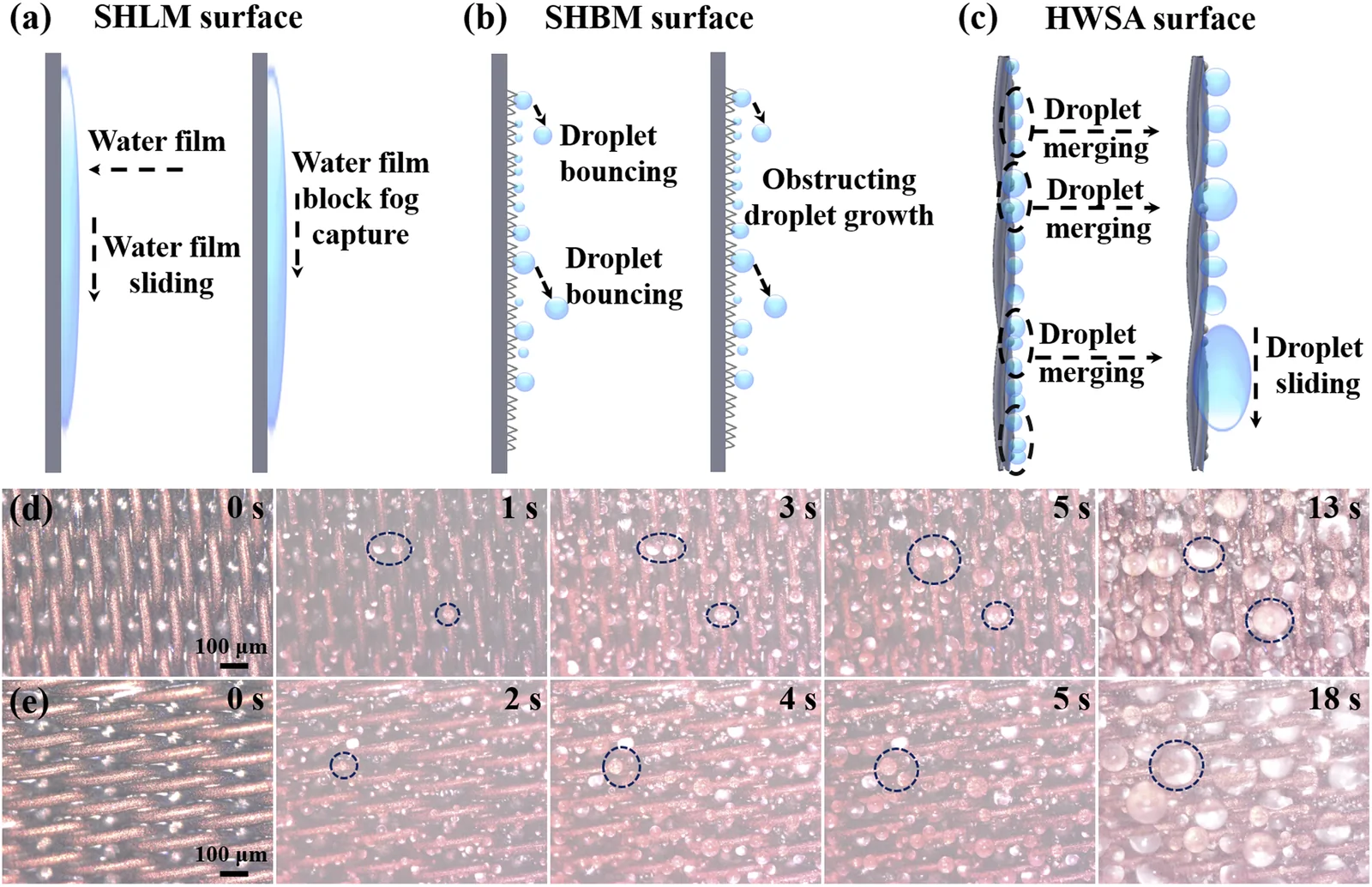
Schematic diagram of droplet migration process on (a) the SHLM surfaces, (b) SHBM surfaces and (c) HWSA surface, respectively. Dynamic behavior of surface droplets (d) vertically and (e) parallelly placed on HWSA.

Detailed droplet migration on surfaces of different wettability is presented in Fig. 5a–c. On SHLM, droplets spread rapidly into a film (Fig. 5a). On the SHBM, small droplets condense but detach slowly (Fig. 5b). On the HWSA surface, droplets are rapidly captured, grow, coalesce, and detach upon reaching a critical size (Fig. 5c), confirming its superior fog collection performance. Additionally, substrate orientation and structural anisotropy also played key roles in collection kinetics. As shown in Fig. 5, droplets nucleate and grow on both vertically and horizontally placed samples. However, on vertically placed samples, droplets slide downward along the grooves (Fig. 5d), whereas on horizontally placed samples, droplets become trapped within the mesh structure with no directional movement (Fig. 5e), impairing growth and detachment. This demonstrates that the woven substrate structure significantly affects collection efficiency.

**Fig. 6:**
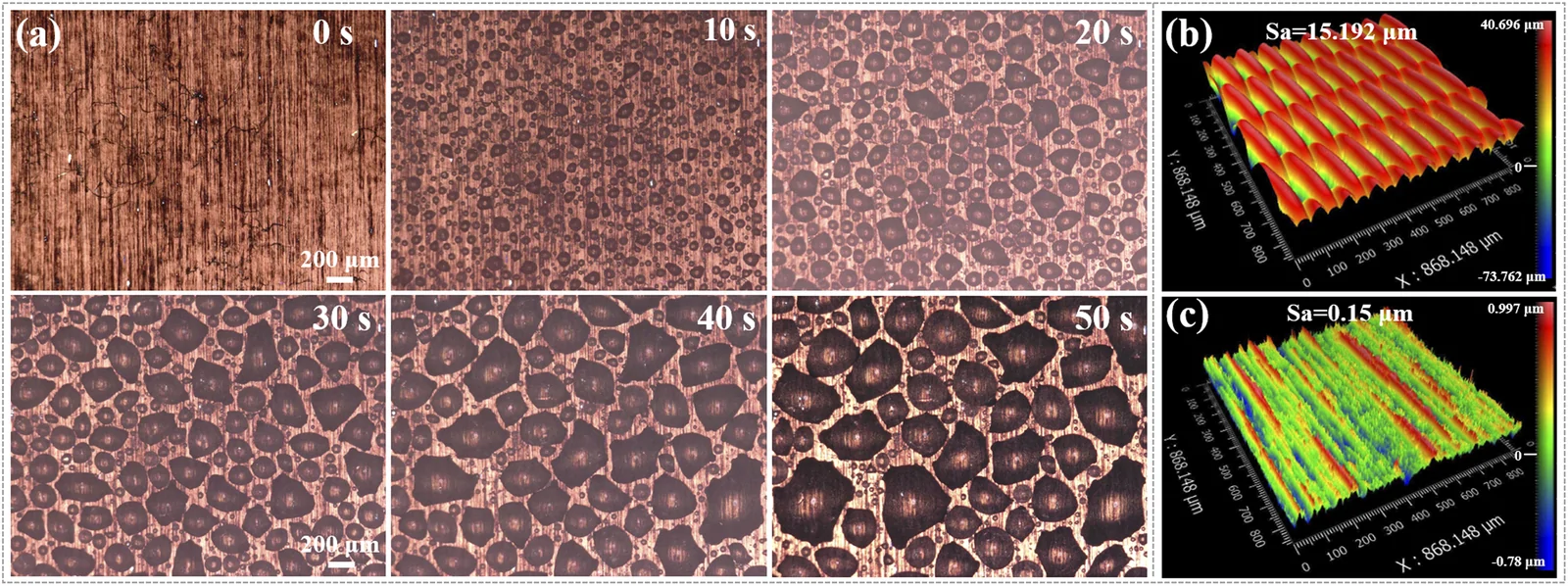
(a) Fog collection process on the HWS surface. Three-dimensional surface topographies of (b) the HWSA surface and (c) the HWS.

**Fig. 7:**
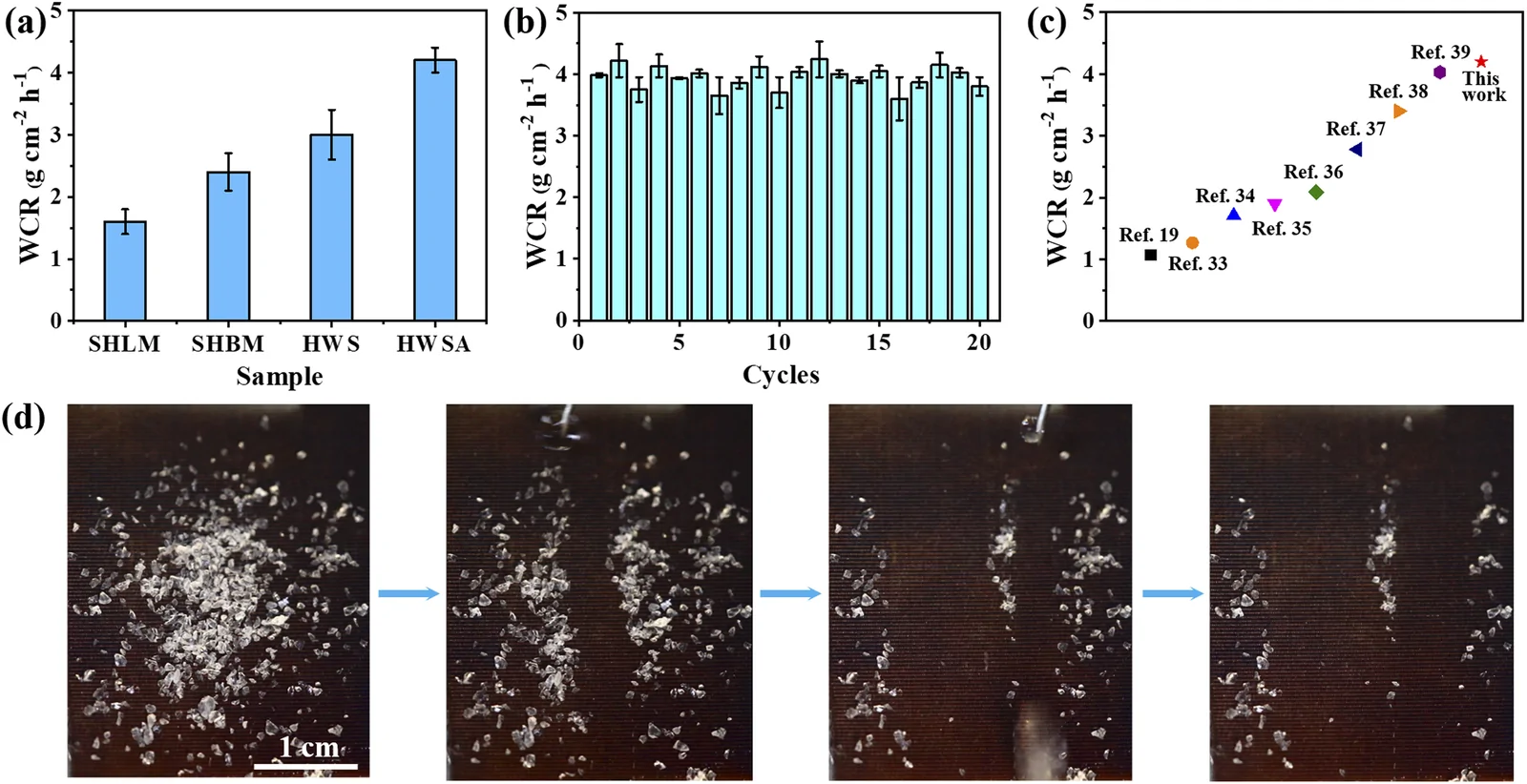
(a) WCR of the different sample. (b) WCR of the HWSA surface over twenty cycles of fog-collecting processes. (c) Comparison of the WCR in this work and other published research. (d) Self-cleaning performance of the HWSA surface.

To further validate the role of substrate-induced anisotropy, we prepared HWS on a flat stainless-steel sheet. As shown in Fig. 6a, droplets condense and grow over time, but they spread broadly and coalesce slowly, ultimately forming irregularly shaped, stationary droplets with no directional motion. Three-dimensional surface profiling (Fig. 6b and c) confirms that HWSA surface possesses a pronounced rough texture, while the flat substrate lacks such anisotropy. Thus, the woven mesh structure itself provides a substantial enhancement to fog collection efficiency. Finally, we compared the fog collection performance of samples with different wettability (Fig. 7a), the HWSA surface exhibits the highest collection rate among all tested surfaces. Given that fog collectors are often deployed in harsh environments, cyclic stability is essential. After 20 consecutive cycles, the HWSA surface showed no significant decline in efficiency (Fig. 7b), and its performance compares favorably with previously reported designs (Fig. 7c).^19,33–39^ Additionally, the HWSA surface exhibits excellent self-cleaning properties (Fig. 7d), which broaden its practical applicability by preventing surface fouling.

## 4 Conclusions

In summary, we fabricated a bioinspired surface integrating hybrid wettability and anisotropy through a simple, low-cost process. The HWSA surface achieves a fog collection efficiency of 4.3 g cm^−2^ h^−1^—substantially higher than that of single-wettability surfaces and flat-substrate hybrid designs. The synergistic combination of hydrophilic droplet nucleation sites and anisotropic hydrophobic grooves enables rapid droplet capture, efficient coalescence, and accelerated detachment, thereby increasing surface refresh rate and overall collection performance. Moreover, the HWSA surface exhibits excellent cycling stability and self-cleaning ability. This facile and scalable fabrication strategy offers a practical solution to freshwater scarcity in arid and water-stressed regions.

## Author contributions

X. B. and Y. L. conceived the research and designed the experiments. Y. L. supervised the research. X. B., Y. C., L. Z., J. C. carried out the experiments. X. B. conducted the reference review and data analysis. X. B. and Y. L. prepared the manuscript. All the authors analyzed the data and discussed the results.

## Conflicts of interest

The authors declare that they have no known competing financial interests or person relationships that could have appeared to influence the work reported in this paper.

## Data Availability

Data will be made available on request.
